# A Computational Platform to Support the Detection, Follow-up, and Epidemiological Surveillance of Mental Health and Substance Use Disorders: Protocol for a Development and Evaluation Study

**DOI:** 10.2196/44607

**Published:** 2023-04-25

**Authors:** Juan Martínez-Miranda, Martha Janet Meza Magallanes, Cándido Silva-Peña, Martha Xitlali Mercado Rivas, María del Rocío Figueroa-Varela, Magda Lidiana Sánchez Aranda

**Affiliations:** 1 Unidad de Transferencia Tecnológica Tepic Centro de Investigación Científica y de Educación Superior de Ensenada Tepic Mexico; 2 Centro de Atención Primaria en Adicciones Servicios de Salud de Nayarit Tepic Mexico; 3 Unidad Académica de Ciencias Sociales Universidad Autónoma de Nayarit Tepic Mexico; 4 Unidad Académica de Medicina Universidad Autónoma de Nayarit Tepic Mexico

**Keywords:** computational platform, mental health, substance use, epidemiological surveillance, digital screening, substance use disorder, suicidal behavior, level of health care, mHealth, substance abuse, addiction, mental health and addiction, addict, IVDU, intravenous drug, opioid

## Abstract

**Background:**

According to the World Health Organization, approximately 15% of the global population is affected by mental health or substance use disorders. These conditions contribute significantly to the global disease burden, which has worsened because of the direct and indirect effects of COVID-19. In Mexico, a quarter of the population between the ages of 18 and 65 years who reside in urban areas present a mental health condition. The presence of a mental or substance abuse disorder is behind a significant percentage of suicidal behaviors in Mexico, where only 1 in 5 of those who have these disorders receive any treatment.

**Objective:**

This study aims to develop, deploy, and evaluate a computational platform to support the early detection and intervention of mental and substance use disorders in secondary and high schools as well as primary care units. The platform also aims to facilitate monitoring, treatment, and epidemiological surveillance ultimately helping specialized health units at the secondary level of care.

**Methods:**

The development and evaluation of the proposed computational platform will run during 3 stages. In stage 1, the identification of the functional and user requirements and the implementation of the modules to support the screening, follow-up, treatment, and epidemiological surveillance will be performed. In stage 2, the initial deployment of the screening module will be carried out in a set of secondary and high schools, as well as the deployment of the modules to support the follow-up, treatment, and epidemiological surveillance processes in primary and secondary care health units. In parallel, during stage 2, patient applications to support early interventions and continuous monitoring will also be developed. Finally, during stage 3, the deployment of the complete platform will be performed jointly with a quantitative and qualitative evaluation.

**Results:**

The screening process has started, and 6 schools have been currently enrolled. As of February 2023, a total of 1501 students have undergone screening, and the referral of those students presenting a risk in mental health or substance use to primary care units has also started. The development, deployment, and evaluation of all the modules of the proposed platform are expected to be completed by late 2024.

**Conclusions:**

The expected results of this study are to impact a better integration between the different levels of health care, from early detection to follow-up and epidemiological surveillance of mental and substance use disorders contributing to reducing the gap in the attention to these problems in the community.

**International Registered Report Identifier (IRRID):**

DERR1-10.2196/44607

## Introduction

### Background

According to the World Health Organization (WHO), health is not merely the absence of a disease, but it is a state of complete physical, mental, and social well-being [1]. Nevertheless, it is estimated that the number of individuals who have a mental disorder worldwide is approximately 792 million, representing 10.7% of the world population. This percentage is increased to 15% when individuals presenting conditions associated with substance abuse are considered [2]. The consequences of mental and substance use disorders are highly negative; in 2010, these disorders constituted 10.4% of the global burden of disease and were the leading cause of years lived with disability among all disease groups [3]. Moreover, these problems have been exacerbated because of the direct and indirect effects of COVID-19 as already evidenced in recent studies [4-8]. Mental and substance use disorders also produce high mortality rates. For example, major depression (the most prevalent mental disorder in the world population) is the main cause of the 800,000 suicides committed every year [9]. Other studies estimate 585,000 deaths only in 2017 associated with the addiction of different substances such as hepatitis C, liver cancer, or cirrhosis [10].

Mental health and substance use problems are also present in Mexico. According to the results obtained from the WHO World Mental Health Survey Initiative, one-fourth of Mexican respondents aged 18 to 65 years and residents in urban areas present with a mental health condition [11]. The most frequent mental disorder was anxiety, followed by other affective disorders such as depression and substance abuse. These results vary according to the sex of the individuals; men reported a more significant number of substance abuse disorders (particularly alcohol) than women. In contrast, women reported more frequent anxiety and affective disorders than men [12]. Similar to the worldwide statistics, in Mexico, the presence of a mental or substance abuse disorder is strongly associated with suicidal behaviors; about half of people with suicidal ideation (48.8%) and two-thirds (65.2%) of those who have tried it at least once have these types of disorders [13]. These percentages highlight the importance of detecting and treating these types of disorders, especially considering a growing trend in the suicide rate from 3.5 suicides per 100,000 inhabitants in the year 2000 to 5.2 in 2017, reaching a rate of 9.3 among the young population (20-24 years old), which represent the second cause of death in this population group [14].

Despite these statistics, mental health care and addiction prevention have been pending issues for public health for years in many countries, and unfortunately, Mexico is not the exception. Although 1 in 4 Mexicans have had a mental disorder at some point in their life, only 1 in 5 of those who have it receive any treatment [15]. Recent studies have revealed that the treatment gaps for severe mental disorders, severe to moderate mental disorders, and substance use disorders in Mexico and other countries across the Americas are 77.4%, 78.7%, and 83.7%, respectively. This gap in treatment is even greater in children and adolescents, reaching up to 86% in Mexico [16]. Some of the main causes of the lack of care for people with mental and substance use disorders in Mexico are the low percentage of resources invested; not enough psychiatric hospitalization units in general hospitals as well as specific services for children and adolescents; the lack of evaluation and treatment protocols for this type of disorder in primary care; and the low rate of psychiatrists, doctors, and psychologists per 100,000 inhabitants [15].

The Pan American Health Organization has proposed different strategies to reduce the gap in attention to mental health problems and addictions in the region. Some of these strategies include the development of national plans, policies, and laws on mental health; the promotion of mental health and the prevention of psychosocial disorders with an emphasis on children and adolescents; decentralization of outpatient mental health services; the need to develop and expand mental health care in primary care; and the need to strengthen the development of national mental health information systems [17]. For the specific case of Mexico, the gradual increase in health spending for mental health and the restructuring of its distribution mode has also been identified as vitally important. Additional recommended actions are strengthening mental health teaching in medical careers, maintaining continuous training for primary care personnel, and increasing society’s participation in establishing solid links with the health sector [15].

Implementing these strategies must have an interinstitutional and multidisciplinary approach allowing for maximum efficiency and does not entirely leave the responsibility to the health sector. In this sense, information and communication technologies are a fundamental tool that would improve the current care model from the phases of detection, early intervention, and continuous monitoring of patients in the community to the creation of information systems for epidemiological surveillance. These computational tools would help in the decision-making and evaluation of the public policies implemented. These digital-based tools would also facilitate a smooth integration between the different levels of health care, offering timely and comprehensive care for people detected with mental health and substance use problems.

One of the main benefits of digital tools for the prevention and treatment of mental and substance use disorders is the increment of access to health care services due to the wide availability of, for example, web-based and mobile apps. Through these apps, adolescents and young adults can be more easily reached and intervened because they are familiar with new technologies or are more technologically literate and mainly involved in using these digital solutions [18]. Examples of these digital solutions and their reported effectiveness can be found in different reviews for both mental health and substance use disorders (see, eg, [19-22]). Moreover, digital platforms also facilitate the screening of substance use disorders and subsequent referral of patients to specialized services [23], expanding and enhancing their treatment [24], or performing analysis for epidemiological studies and supporting clinical decision-making [25].

### Objective

This protocol describes the implementation and evaluation of a computational platform to support the early detection and intervention of mental and substance use disorders at the scholar community level and in primary care units and facilitate monitoring, treatment, and epidemiological surveillance at the second level of care. Three specific objectives lead to the development of the computational platform:

1. Facilitate the early detection of symptoms and risk factors associated with mental and substance abuse disorders through a web-based module for the screening of symptoms and risk factors related to mental and substance abuse disorders in the scholar community.
2. Support the follow-up process and the surveillance of detected cases through the module to collect, process, and visualize the information of individuals screened and under treatment. Integrating the collected information from the screening, the first contact in primary care, and the treatment and follow-up provided at the secondary care level will optimize the whole care process of individuals with mental health and substance use disorders.
3. Support early interventions and the continuous monitoring of patients under treatment. These applications aim to prevent and minimize the appearance of risk behaviors or promote and motivate the change of these behaviors to maintain an adequate state of health.

### Consortium

The development and evaluation of this computational platform is a collaborative effort between 4 institutions located in the state of Nayarit, Mexico. The Centre for Scientific Research and Higher Education at Ensenada is a public research center and is the project’s coordinating institution. Moreover, it contributes to the technical development of the computational platform integrating artificial intelligence and human-computer interaction techniques in the different modules of the platform. The Health Services of Nayarit is the local government institution responsible for the health services in Nayarit. The clinical specialists of this institution contribute to defining the screening, intervention, and follow-up processes supported by the computational platform. The specialists of this institution are also involved in the deployment and evaluation of the platform involving the patients at different stages of the detection and follow-up processes. The Public Education Department of Nayarit is the institution that coordinates with the educative institutions in Nayarit. The participants from this institution contribute to facilitating access to the selected secondary and high schools for the early detection process. They will also contribute to evaluating the platform’s impact on the scholar community. The Autonomous University of Nayarit is the fourth participant institution. The researchers of this institution contribute to the definition of the functional and user’s requirements, participate in the detection and follow-up process of students from high schools, and evaluate the platform.

## Methods

### Modules of the Computational Platform

The achievement of the specific objectives guides the identification of the different functionalities that the computational platform provides to all the users involved: clinicians, teachers, and individuals at risk or detected with mental health and substance use disorders. Three main modules make up the proposed computational platform:

1. The module to support the screening of symptoms and risk factors. This module implements a set of standardized and validated instruments to detect individuals at risk. The screening module can be accessed from the scholar community (secondary and high schools) and primary care units. Based on the screening results and when necessary, this module provides personalized suggestions to refer detected individuals to the different services offered by the health sector. Moreover, the personnel responsible for the prevention and early detection processes (clinical specialists and school staff) will be informed about those individuals at risk to continue with the follow-up process.
2. The module to facilitate the referral, intervention, treatment, and epidemiological surveillance of the detected cases of mental health and substance use disorders. The specialists will use this module at the primary and secondary care to aid in the management and monitoring of individuals under treatment in both care levels. All the collected information from the detected cases is processed to generate graphical and statistical reports at the aggregated level for epidemiological surveillance purposes.
3. The module to support early intervention and continuous monitoring. This component includes the integration of web-based or mobile apps used by patients or individuals at risk. Our research group has experience in developing these types of apps with advanced interaction and artificial intelligence techniques such as embodied conversational agents and serious games (eg, [26,27]). Together with clinical specialists of our research consortium, we are assessing the development of the most appropriate strategy according to the characteristics and conditions of the target population.

**Figure 1:**
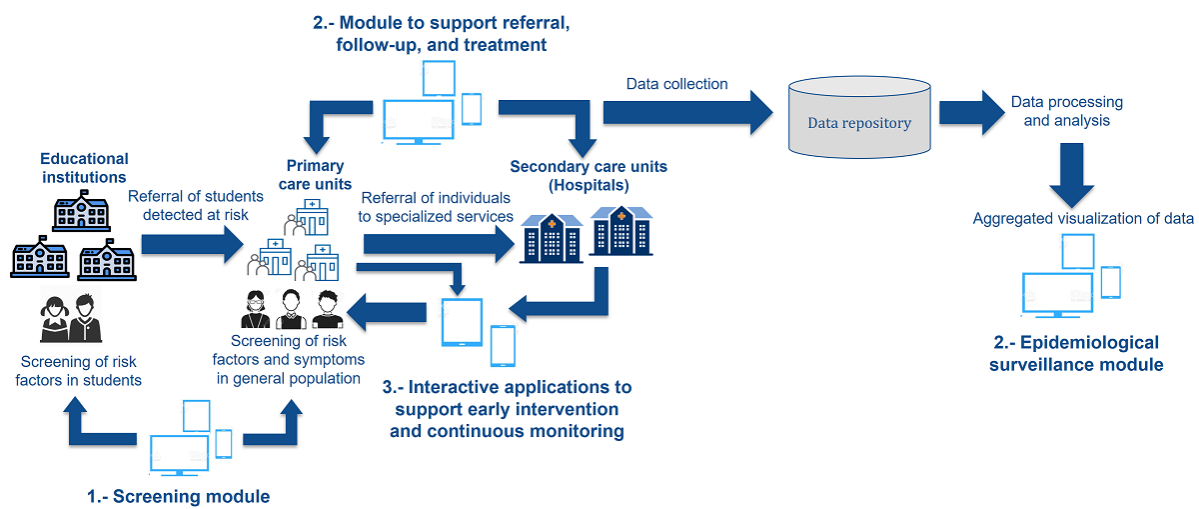
Functional architecture of the computational platform.

The visual representation of the components and deployment of the computational platform to be developed is shown in Figure 1. Each of the computational platform modules is accessible by different users according to the phases of detection, intervention, monitoring, and epidemiological surveillance. The screening module can be accessed from any device, and the implemented questionnaires can be answered directly by the individuals to assess with the guidance of a specialist. This process makes it easier to reach a greater number of people and can be applied anywhere: schools, primary care units, or emergency areas of hospitals. Depending on the obtained results, individuals’ referral to primary or secondary health care services is also supported by the platform.

The collected information during the screening process is sent to the primary care units facilitating an early identification of those individuals detected at risk who would require a complementary medical assessment. All these detected individuals are asked to attend the primary care unit. When the person arrives at the primary care unit, the computer platform will facilitate recording the data and the complementary assessment and, if required, referring to the second level of care to receive specialized treatment. The information collected during this process is stored in a central information repository available to all the specialists involved in the care process. Additionally, the analysis and processing of the collected information in an aggregated and anonymized fashion is performed for epidemiological surveillance purposes.

Finally, as part of the computational platform, web- or mobile-based apps are considered to support early intervention or complement psychotherapeutic treatment through continuous monitoring. Examples of these apps are serious games to help in the prevention of alcohol and drug consumption, or an interactive app for the monitoring of, for example, emotional states, behavioral activities, or frequent thoughts would help to assess the evolution of the patient’s condition. The information collected from these apps is integrated into the patient’s electronic file stored in the data repository for subsequent consultation by the specialist. This information has proven helpful for specialists as it makes it easier to get relevant details about a patient’s condition during interconsultation periods.

### User-Centered Design

One of the main issues when developing computer-based systems within the health sector is the failure to adopt these solutions as part of the activities within clinical practice. Scientific evidence in the area agrees that technological factors do not cause this problem, but it is caused by the systematic lack of consideration of human and sociotechnical factors from the target users, especially during the design and implementation stages of these solutions [28]. The lack of adequate involvement of end users leads to a poor understanding of the functional requirements, suboptimal usability of these systems, and an exacerbated resistance to the change and adoption of these systems. To minimize these risks and maximize the acceptability (and consequently the effective adoption) of the proposed computational platform, we follow a user-centered design methodology, which is widely used in developing software and technological solutions. The most relevant characteristics of this methodology are as follows [29]:

1. Emphasize the identification of the needs and requirements of end users that the technological product must cover.
2. Identify and understand the context in which the technological product will be implemented.
3. Design the technological solution based on these real needs of the users and the identified context and not on preconceived ideas of the developers or established and rigid guidelines.

Thanks to the participation of the different institutions in the consortium, the adoption of the user-centered design methodology will be facilitated by involving all the direct and indirect users of the platform: clinical specialists at the different levels of care, personnel from the educational institutions, and people at risk or under treatment of mental health and substance use disorders. All these users collaborate from the beginning of the project and throughout its final deployment and evaluation.

### Workplan

**Figure 2:**
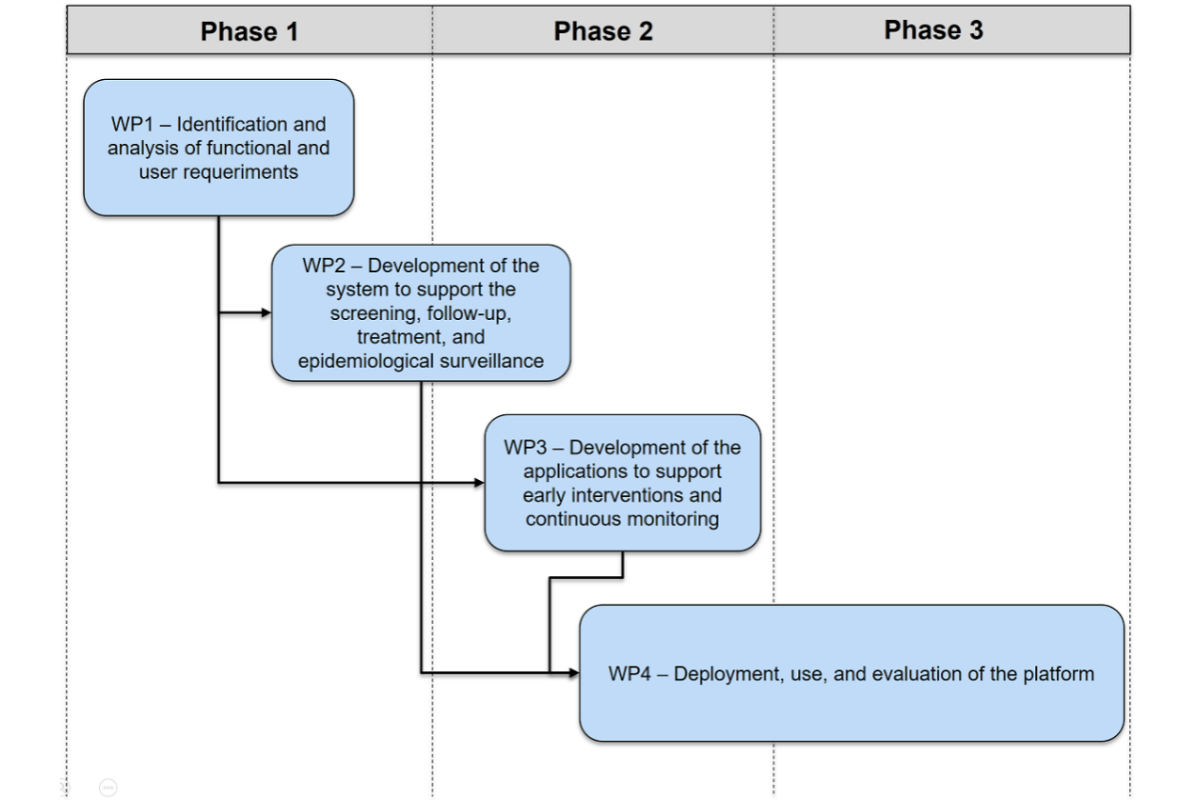
Overview of the work packages defined for the development of the computational platform. WP: work package.

The designed workplan for the development of the computational platform considers the 4 work packages (WPs), as illustrated in Figure 2, distributed into 3 annual stages: WP1 identification of the functional and user requirements; WP2 development of the system to support the screening, follow-up, treatment, and epidemiological surveillance; WP3 development of the apps to support early interventions and continuous monitoring of patients; and WP4 deployment and evaluation of the platform.

The activities defined in WP1 are focused on identifying functional requirements for every module of the computational platform. Examples of these requirements include the selection of all the standardized and validated—in the Mexican population—instruments used in the screening process. Depending on the characteristics of the individuals (eg, the age) to screen, the system will show the adequate set of instruments (eg, the Problem-Oriented Screening Instrument for Teenagers [POSIT] [30]) that the specialist selects to detect the type of mental health or substance use problem. This WP1 also define the data to collect from each screened person, the recommendations provided according to the screening results, and the process to facilitate the follow-up and treatment of the individuals detected at risk in the subsequent attention at primary and secondary health units.

Other activities in this WP1 include the identification of the user and functional requirements for the development of the apps to support early interventions and continuous monitoring. Different focus groups with the target users will be considered to identify the specific objectives of these apps. Examples of these apps are to support (universal or particular) prevention of substance use through a gamified app for teenagers or an app for the remote and continuous monitoring of individuals with a mental health disorder (eg, depression and anxiety) based on cognitive behavioral therapy contents. Moreover, in this WP1, the selection of the scholar community forming the study sample for the screening process is performed. The schools’ selection follows a set of agreed criteria (eg, geographical location, the quantity of student population, number of cases previously detected, or students’ sociodemographic characteristics). Sensibilization sessions will be carried out with the personnel of the selected schools to inform them about the characteristics and objectives of the project.

WP2 and WP3 contain all the activities for the design and development of the different modules of the computational platform. The design, development, and testing process are iterative and follow the user-centered design methodology. Based on the information identified in WP1, each module is designed for the collection and processing of the data at different stages of the health care process: screening, intervention, treatment, monitoring, and epidemiological surveillance. Focus groups with different types of users have been considered since the first iterative design process. The result of these 2 WPs is the integration of the different modules forming the computational platform ready for its deployment and use in the selected educative institutions and health units.

The last WP4 includes all the activities required for the deployment, use, and evaluation of the computational platform. The deployment is planned to be gradual along the project's final year in the selected schools and health units. Training sessions will be carried out with the personnel of all the institutions where the platform is deployed. All technical issues that could emerge during the platform use are collected for the functional improvement of each module. Moreover, the collected information from the use of the platform will be processed for usability, acceptability, and impact evaluation in the health care process. Activities for the diffusion and dissemination of the project and the obtained results are also included in this WP4.

### Recruitment

The study participants from whom information will be collected in the computational platform are of two types:

1. Participants in the scholar community (secondary and high schools) will be screened using a set of validated and standardized questionnaires to detect symptoms and risk factors associated with mental or substance abuse disorders.
2. People attending a health unit because of the presence of a mental or substance abuse problem. These participants can be 12 years and older and of both sexes. Personal data and relevant information related to the follow-up process will be recorded, for example, reference data between the different levels of health units, personal data, and notes of diagnostic evolution that will form their electronic clinical file.

For the screening process in the scholar community, the participants are the students (males and females aged 12-18 years) of the selected schools for the study sample. In coordination with the school personnel in each school, the health professionals select the specific groups for the screening. Explicit informed consent is asked from each student and their parents or legal guardians. Then, the screening instrument (the POSIT questionnaire) is applied to all the students who provide their explicit authorization. The teacher or the health specialist provides the instructions to respond to the questionnaire and is available to answer any questions during the process. The screening session is expected to last around 45 minutes for each group.

For the people who attend primary care units to receive medical attention, the doctor who receives them will inform them that the screening is part of the diagnostic assessment process. If the patient is a minor, an explicit authorization to carry out the screening process must be provided by the minor and by the accompanying adult. The duration of the session will be the time of the medical visit that the specialist decides.

In both groups, depending on the obtained results provided by the computational platform, health personnel identify those individuals who require a follow-up intervention delivered by a specialist and are referred to the corresponding health care unit.

The recruitment of participants who will use the apps to support early intervention and continuous monitoring will be carried out by the specialist responsible for their treatment. In the case of minors, explicit authorization from their parents or guardians will be asked before the provision of the application. In all the cases, the following exclusion criteria are considered:

1. A diagnosis of schizophrenia, psychosis, or borderline personality disorder
2. Not having a digital device to execute the app
3. Any disability that makes the individual unable to use a digital app
4. Requirement to be under personal surveillance due to current symptoms associated with a mental health or substance use disorder.

### Data Collection

The collected data from all the participants include personal and sociodemographic data such as age and sex. For participants of secondary and high schools, the answers to each of the POSIT’s questions and the final score are also collected. For the individuals recruited in primary care units, the results obtained from different instruments to assess their condition (eg, the Alcohol, Smoking, and Substance Involvement Screening Test questionnaire [31], a suicide risk measure [32], or the Hamilton Anxiety Rating Scale [33]) are also collected. The epidemiological surveillance module uses the final scores and the corresponding interpretation of these instruments to visualize the type of risks or disorders detected in schools and health care units. Moreover, all the information produced during the follow-up process is also collected to support the specialists with the whole treatment of the individuals who require the services of a secondary level of care. The data generated during the use of the web- or mobile-based apps by the participants are also collected, and the information is used for a continuous patient evolution monitoring. These data include, for example, the current mood, frequent—rational or irrational—thoughts, or the planning and development of specific activities.

### Storage and Security of the Information

The data collected at schools and health units will be sent via the internet, using a secure data transfer protocol (https) to a central information repository. All data will be textual, and no multimedia files such as photographs and audio or video recordings are included. The data repository is located in the central offices of the Health Services of Nayarit, with the appropriate access and security protocols and the necessary data backup processes. The collected data are accessed through a username and encrypted password through the developed computer platform. The access depends on the user’s role; the personnel who analyze the screening results have access to consult the data that allow the person screened to be located and recommend the appropriate follow-up. The specialists responsible for clinical treatment have access to all the information collected in the electronic health record created for each patient. Finally, the users of the epidemiological surveillance module have access to the aggregated and anonymized data to know the incidence and prevalence of the different mental and substance use disorders registered in the computer platform.

### Evaluation

According to the WHO, the evaluation of a digital health intervention involves the measures taken and analysis performed to assess two main issues: (1) the interaction of users or a health system with the digital health intervention strategy or (2) the changes attributable to the digital health intervention [34]. The assessment of the interaction between the platform and the target users will be performed through a mixed methods approach [35] with an explanatory design study using validated and standardized questionnaires to get information about the usability, acceptability, and perceived usefulness from the users of the different modules of the platform: health specialists, teachers and personnel of the educational institutions, and individuals at risk or patients. Existent validated instruments will be applied to a representative set of users after the deployment and use of each module. Qualitative information will be gathered throughout focus groups with a Logical Framework Approach.

For all web-based modules, the System Usability Scale and the Technology Acceptance Module will be provided to the users to rate the platform modules’ usability, acceptance, and ease of use. The System Usability Scale consists of a 10-item questionnaire with 5 response options for respondents: from *strongly agree* to *strongly disagree*. This instrument allows evaluating a wide variety of products and services, including web-based health apps [36,37]. The Technology Acceptance Module measures the constructs of perceived usefulness, ease of use, and user acceptance of the technological product in the participant’s workflow by explaining the user’s “behavioral intention” with the system. This instrument is based on the theory of reasoned action [38], a psychological approach that seeks to explain behavior, and has also been widely applied in eHealth systems [39]. If any of the platform modules is deployed through a mobile device (eg, the apps for early intervention and continuous monitoring of patients in the community), a complementary instrument such as the Mobile App Rating Scale will be used to evaluate these modules. The Mobile App Rating Scale is a simple, objective, and reliable tool for classifying and assessing the quality of mobile health apps [40].

The changes in the health care process attributable to the platform will be assessed through a quantitative study by comparing the population coverage achieved with the computational platform in relation to current and historical data. Moreover, a Logical Framework Approach will be adopted to identify individual, social, and contextual factors affecting the mental and substance use health care process at primary and secondary levels. This approach has previously used to identify relevant factors and characteristics in different health care processes such as emergency care [41], hospital-based health care services [42], or rural health missions [43]. The Logical Framework Approach will be implemented through focus group sessions involving specialists and asking to identify the main problems (barriers) and solutions (facilitators) each participant faces during the different stages of the health care process. These sessions will be carried out before the platform’s implementation and then after its deployment and use by the different specialists and patients or individuals at risk. The collected information from these sessions will allow the identification of specific problems that could be minimized or removed due to the different functionalities of the platform. The detected changes will help to assess the impact of the digital intervention along the process: from the early detection in the scholar community to the follow-up and treatment of individuals with mental health and substance use disorders in primary and secondary health units.

The evaluation results will be disseminated among the community and public health policy makers, emphasizing the platform advantages of adopting it as part of a public health program. Moreover, these results will lead to the identification of lessons learned during the deployment and use of the platform providing valuable recommendations to maximize the benefits for the attention of mental health and substance use disorders in other regional and national health systems.

### Ethics Approval

The study has obtained research ethics approval from the institutional review board of the Health Services of Nayarit (CEBN/04/2020). Participation will be voluntary, and informed written consent or digital consent will be obtained from participants and from parents of those participants younger than 18 years. The participants will be informed about the confidentiality of their answers to the applied questionnaires. All identifiable information will not be accessible, except for the clinicians who will provide formal care services to the participants. Participants’ data will not be identifiable in any publication or report. No monetary compensation will be offered to the participants.

## Results

The project has been running since July 2021 and is intended to complete in June 2024. An initial version of the modules to support the screening and follow-up treatment processes has been developed, and the initial deployment and testing of these modules in a school sample of 10 secondary schools and 10 high schools located in the city of Tepic has started during the second semester of 2022. Currently, 6 schools have used the screening module implementing the POSIT questionnaire, and as of February 2023, a total of 1501 students have undergone screening. The referral of those students presenting a risk in mental health or substance use to primary care units has also started. The second stage includes the development of the interactive apps to support early intervention and continuous monitoring and the epidemiological surveillance module. The use and evaluation of the complete platform are the main activities for the final year of the project, and the obtained results will be disseminated between the research community, health specialists, and the general public.

## Discussion

### Principal Findings

This protocol describes the development and evaluation of a computational platform to support the detection, intervention, treatment, and epidemiological surveillance of mental health and substance use disorders in the community and primary and secondary health units. Three main modules form the computational platform: (1) a module to support the screening of symptoms and risk factors; (2) a module to facilitate the referral, intervention, treatment, and epidemiological surveillance of the detected cases; and (3) interactive apps to support and complement the provision of early intervention and continuous monitoring. Some modules of the platform are currently under development, and their complete deployment and evaluation are expected at the end of 2024.

It is expected that the proposed platform will contribute to a better integration of the involved institutions, professionals, and processes in the attention to mental health and substance use disorders. The early detection of risks in the scholar community will contribute to a timely referral to formal health services, minimizing the future chronicity of these disorders and the subsequent associated problems, including suicides. The functionalities of the computational platform to support the follow-up and monitoring of patients under formal treatment, as well as the generation of epidemiological information on the incidence and prevalence of these disorders, will help to perform qualitative and quantitative analyses to target better public policies and strategies implemented as responses to this problem, as well as their evaluation.

### Limitations

This study has some limitations that require consideration. First, the recruitment of participants aged 13 to 17 years is restricted to those attending secondary and high schools. In Mexico, there are a significant number of individuals within this age range who are not in school and are at high risk of substance use. Second, the platform will only be used by clinicians belonging to the public health services of Nayarit. However, there are various public health systems in Mexico, even within the same city or region, and individuals identified with risk factors or symptoms will be referred to the corresponding institution where they receive their health services. Therefore, individuals referred to health units outside of the public health services of Nayarit will not be followed up or treated with the platform. It is expected that positive results, such as a better integration between the different levels of care, from early detection to follow-up and epidemiological surveillance, will encourage the adoption of the platform by other public health systems.

### Conclusions

Digital-based solutions designed to support health care processes have the potential to increase the accessibility of the general population to health services, mainly due to the wide availability of, for example, web-based and mobile apps. This is particularly important for mental health and substance use disorders, where treatment gaps reach percentages above 75% in Mexico. The development, deployment, and evaluation of the computational platform proposed in this study have the potential to contribute to the reduction of this treatment gap by improving the integration and exchange of information between the different specialists and health care units involved in the different processes of attention: from the early detection of these health problems in the community to the analysis and visualization of the identified cases with epidemiological surveillance objectives.

## Abbreviations

POSIT: Problem-Oriented Screening Instrument for Teenagers
WHO: World Health Organization
WP: work package

## Data Availability

Data are not publicly available but can be obtained via contacting the corresponding author.
